# Delayed Onset of Subclavian Artery Pseudoaneurysm With Brachial Plexus Compression Following Gunshot Wound Injury

**DOI:** 10.7759/cureus.22457

**Published:** 2022-02-21

**Authors:** Thomas Huang, Clayton W Armstrong, Geoffrey D Panjeton

**Affiliations:** 1 Anesthesiology and Critical Care, Saint Louis University School of Medicine, Saint Louis, USA

**Keywords:** vascular injury, spinal cord injury, upper extremity trauma, subclavian pseudoaneurysm, brachial plexus injury

## Abstract

Early diagnosis of brachial plexus injuries is crucial to prevent long-term morbidity and improve outcomes. We present a unique case of delayed onset of brachial plexus compression two months following a traumatic gunshot injury causing multiple injuries including a T1 vertebral body comminuted fracture and pneumothorax. The patient experienced significant pain and progressive neurological examination changes during follow-up visits, and thus duplex ultrasound and computed tomography (CT) angiography were performed, which demonstrated a left subclavian artery pseudoaneurysm. This was managed operatively by evacuation and interposition bypass. Injuries to the cervical and upper thoracic spine are complex, and when patients present with new-onset neurological findings, axillary swelling, or significant uncontrolled postoperative pain, secondary complications should be suspected. Patients at a high risk of vascular reinjury should be routinely monitored at follow-up to prevent the development of progressive neurological damage to the brachial plexus.

## Introduction

Subclavian vessel injuries are extremely precarious as they can cause devastating neurological injury due to proximity to the brachial plexus [1]. The mechanism of injury often involves either penetrating or blunt trauma to the artery. Identifying and addressing the root cause of the injury early is crucial to the restoration of function. Traumatic subclavian artery injuries were poorly studied until World War I [2,3]. The current literature on subclavian artery injury predominantly consists of older patients with shoulder dislocations from traumatic falls [4]. Reports of delayed onset brachial plexus injury due to any form of trauma are similarly sparse [5,6]. Beyond falls, Murata et al. reported a single case of a compressive hematoma developing two days in a teenager after a motorcycle accident [7]. This case report describes a patient who developed a delayed onset brachial plexus injury due to a subclavian artery pseudoaneurysm following multiple gunshot wounds.

## Case presentation

**Figure 1:**
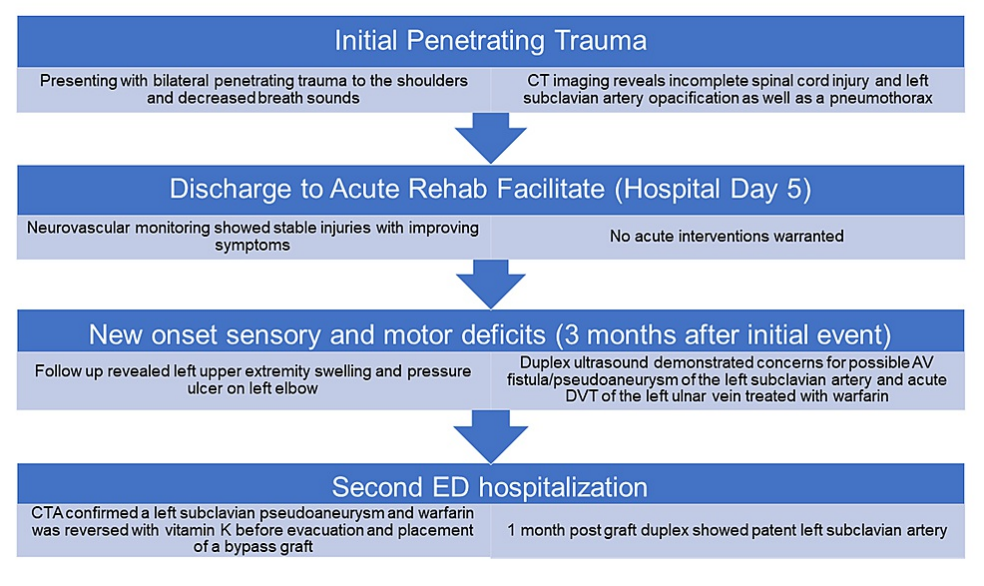
Patient History. CT, computed tomography; LOS, length of stay; AV, arteriovenous; DVT, deep vein thrombosis; CTA, computed tomography angiography

**Figure 2:**
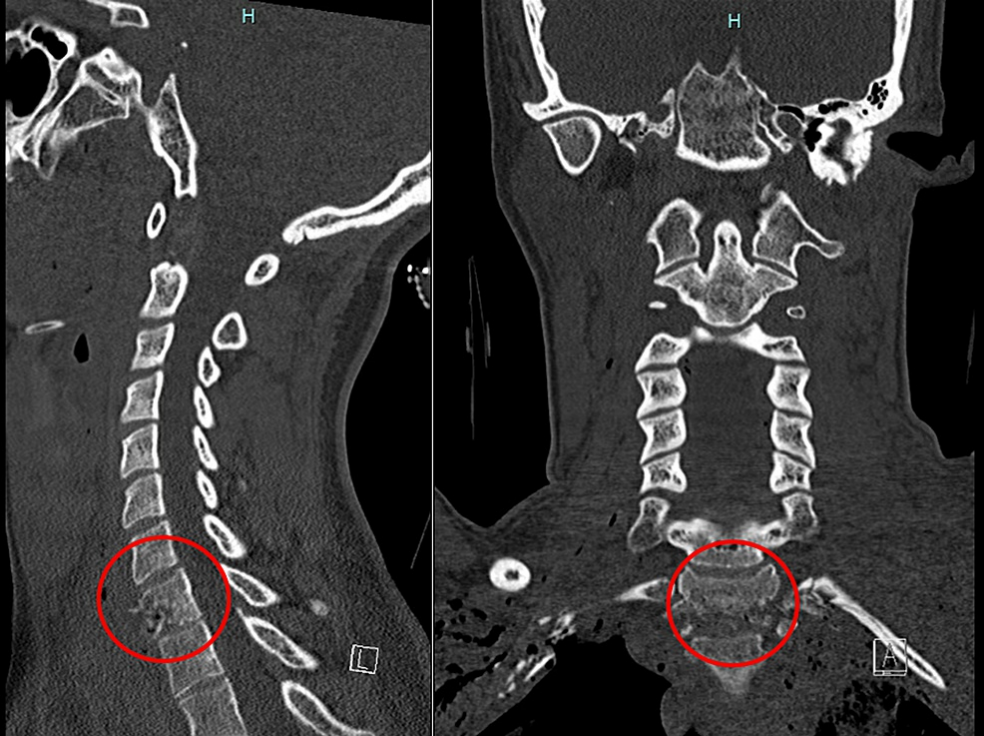
Sagittal and coronal CT of the cervical and thoracic spine showing a comminuted T1 fracture on the day of the trauma demonstrating an incomplete spinal cord injury (circled).

**Figure 3:**
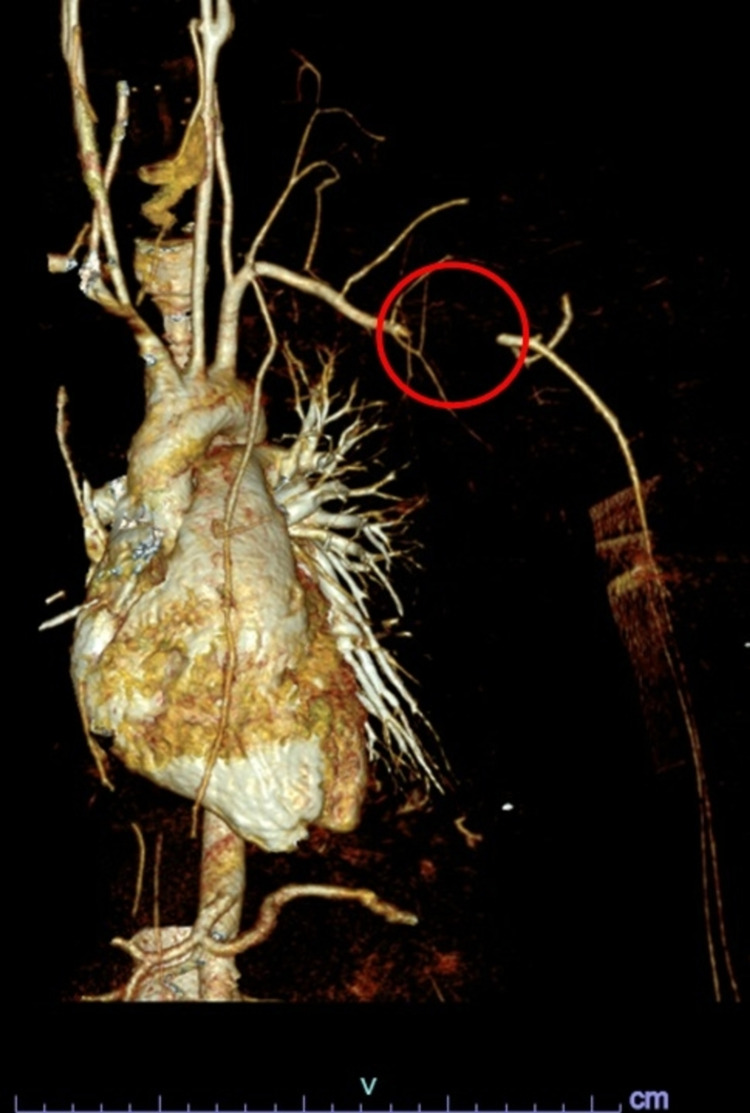
Three-dimensional CT angiography after the initial trauma showing abrupt loss of opacification of the left subclavian artery for 2.9 cm in length and then distal reconstitution of the left subclavian artery (circled).

A 20-year-old female was brought to the emergency department after sustaining multiple gunshot wounds (Figure 1). Her past medical history included a motor vehicle collision six months prior to presentation. On arrival, the patient was in acute distress due to findings of gunshot wounds to bilateral shoulders and decreased breath sounds on the right side. The patient was stabilized in the emergency department following resuscitation with a transfusion of 1 unit of packed red blood cells and a chest tube for the management of a pneumothorax. Physical examination of the spine revealed tenderness but no visible wounds or other palpable deformities. Computed tomography (CT) imaging revealed a comminuted T1 vertebral body fracture with multilevel pneumorachis (Figure 2). This was interpreted as a T1 vertebral body fracture and incomplete spinal cord injury. No acute surgical management was indicated since there was no evidence of spinal cord compression, and the patient was placed in a cervical thoracic orthosis. Chest CT with angiography revealed abrupt loss of opacification of the left subclavian artery for 2.9cm in length and then distal reconstitution, which was concerning for vascular injury (Figure 3).

**Figure 4:**
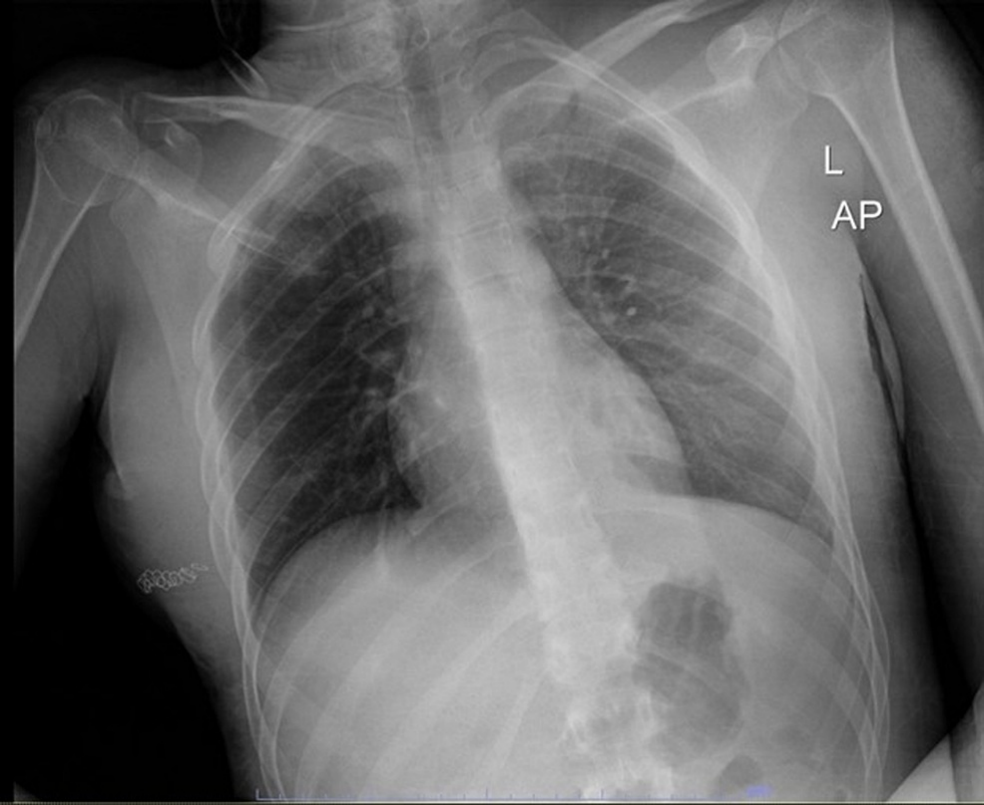
X-ray of the chest three days post-trauma. Note bilateral first rib fractures.

The patient was admitted to the intensive care unit and on hospital day 2. X-ray imaging demonstrated resolution of the pneumothorax. Additionally, the patient was started on a daily dose of aspirin 81mg. On hospital day 3, bilateral first rib fractures were noted on X-ray (Figure 4). Neurovascular monitoring revealed a wrist brachial index of 0.58 in the left radial artery and 0.64 in the left ulnar artery with adequate collateral circulation. Daily subsequent monitoring was reassuring, and findings did not warrant acute surgical intervention. The remaining of the hospital course was unremarkable. The patient was discharged to an acute rehabilitation facility five days after admission in stable condition. At the acute rehabilitation facility, the patient received 45-90 minutes of intense occupational therapy five days a week for three weeks to increase independence for safe discharge to a less restrictive environment.

Follow-up physical examination at one month post-injury was complicated by severe pain, which limited movement and caused significant emotional distress. At the three-month follow-up appointment with her primary care physician, the patient demonstrated new-onset decreased motor strength with a flaccid hand in extension and intact sensation; additional findings included left elbow pressure ulcer, swelling in the left axillary region, and increased pain. Presentation was concerning for possible deep vein thrombosis (DVT), which prompted a venous duplex ultrasound evaluation, which revealed a left ulnar vein thrombosis that was promptly treated with warfarin.

**Figure 5:**
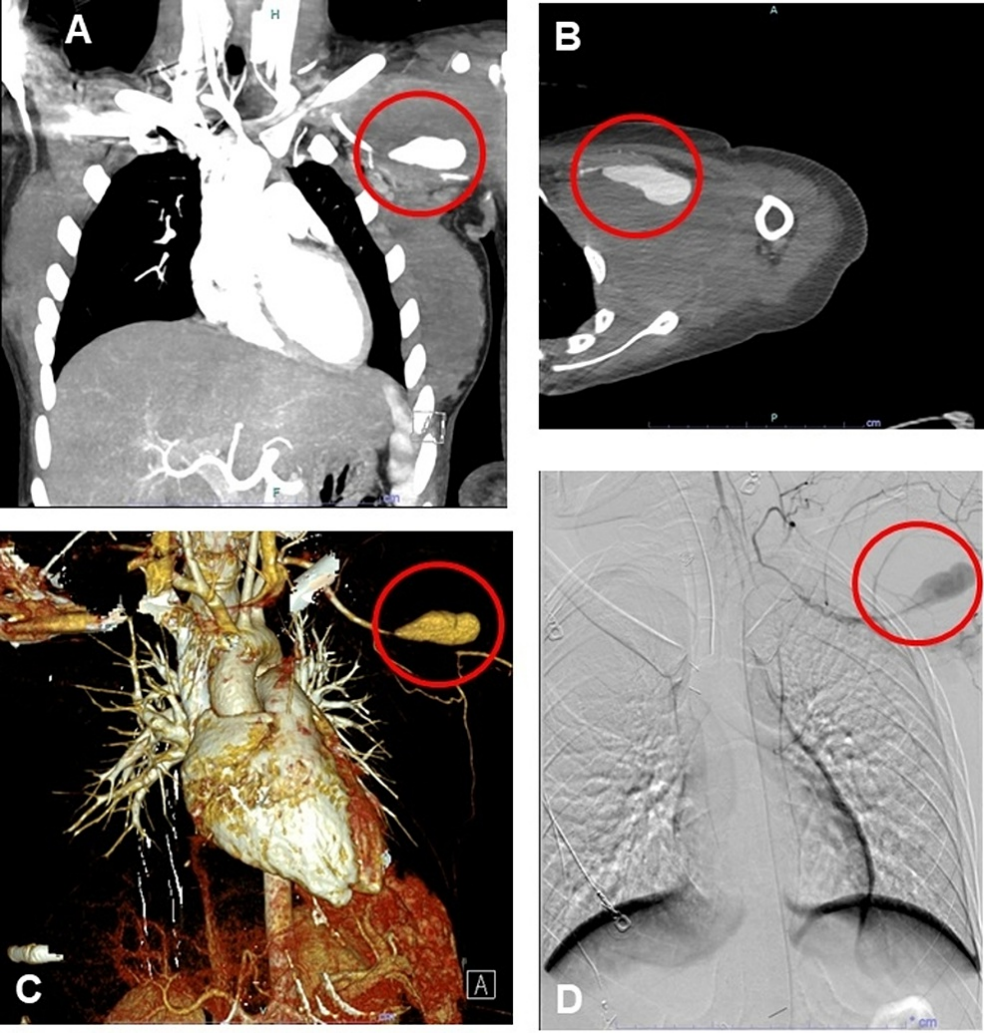
Imaging of the left subclavian artery pseudoaneurysm (circled) measuring 5cm x 1.8cm compressing the brachial plexus through various modalities three months after the initial traumatic event. (A) CT angiography of the thorax. (B) CT angiography of the left upper extremity. (C) Three-dimensional reconstruction of the angiography. (D) Intraoperative fluoroscopy of the left subclavian artery.

The patient presented to the emergency department four days later due to increasing pain. CT angiography revealed a focal narrowing of the left proximal axillary artery associated with a large pseudoaneurysm in a distal segment of the left subclavian artery measuring 5cm x 1.8cm with compression of the brachial plexus (Figure 5). Vitamin K was given to reverse the warfarin before an interposition bypass graft was performed. Physical therapy and occupational therapy recommended home healthcare upon discharge; however, insurance restrictions did not allow for this. On postoperative day 3 (hospital day 5), the patient was anemic and received a transfusion of packed red blood cells. She was later started on a course of apixaban 5mg twice daily for DVT prophylaxis and discharged home on hospital day 6.

One month after the decompression of the pseudoaneurysm and interposition graft, the patient continues to have delayed healing of the left elbow pressure ulcer. Physical examination revealed significant motor deficits in her arm. Further examination demonstrated intact palpable pulses, and left arterial duplex ultrasound of the upper extremity showed a patent left subclavian artery bypass graft with no evidence of stenosis. Prognosis of motor recovery is unclear given the duration of injury and comorbid incomplete spinal cord injury.

## Discussion

Traumatic cervical and thoracic spinal cord injuries are complex, and complications such as brachial plexus injuries can occur. Subsequent patient evaluation should consider possible damage to the adjacent structures including vascular pathology that can damage the brachial plexus. In patients with sustained orthopedic trauma, psychiatric conditions such as post-traumatic stress disorder, depression and/or anxiety, musculoskeletal disability, and pain have been observed to be significantly increased at eight months post-traumatic event [8,9]. This can complicate the assessment of patients for potential post-trauma sequelae. A high index of suspicion is needed to ensure that alternative pathologies are not masked.

Patients with subclavian vessel injuries can present with neurological deficits or decreased and even absent pulses in the affected extremity. Other suggestive features of vessel injury include overlying bruits and ipsilateral clavicle or rib fractures. In a prospective study by McKinley et al., approximately 25% of the 260 patients with subclavian artery injuries did not have prominent symptoms and were often missed by physical examination alone [2,3,10]. Ankle-brachial index (ABI) can be normal in these patients due to collateral circulation in the upper extremity. In patients with comorbid conditions, pain despite doses of analgesics or out of proportion to the examination warrants further investigation [3].

Previous case reports involving blunt trauma to the shoulder demonstrated early concerning signs including swelling, decreased sensation in a dermatomal distribution, decreased muscle strength, cold extremities, and decreased or absent palpable pulses, with one patient demonstrating a progressive anemia [7,11,12]. In a patient with a fall while playing tennis, symptoms progressed from ecchymoses on day 1 to progressive arm weakness without paresthesias on day 2 and eventually hospital admission on day 6 due to abnormal neurological findings similar to the aforementioned case reports [5]. Watanabe and Matsumura reported a case where a delayed-onset brachial plexus injury occurred due to a subclavian artery pseudoaneurysm formation after clavicle fracture in a patient who sought medical attention four months after the trauma with reported pulsatile swelling in the supraclavicular region and pain radiating down the right upper extremity [6].

Initial diagnosis could be assessed with color Doppler ultrasound. Ultimately, angiography (MRI or CT) is indicated for vascular injury that requires surgical intervention [3,13]. In our patient with a subclavian artery injury and bilateral first rib fractures, concern for brachial plexus injury should be high and prompt further monitoring at future visits.

One limitation of this case report is an unclear timeline of pseudoaneurysm progression due to intermittent outpatient follow-up. The progression of a potential pseudoaneurysm is variable. The most practical recommendation would be extensive patient education on concerning signs and symptoms and frequent complete neurological examinations that lead to prompt follow-up imaging. Additionally, due to the intermittent follow-up and complicated patient presentation, the timeline and extent of compression on the brachial plexus is unclear.

In penetrating traumatic cervical or thoracic spinal cord injuries, clinicians should have a high index of suspicion for further vascular or neurological compromise. These patients can have multiple confounders that decrease the sensitivity and specificity of physical examination alone. Accessible imaging modalities at follow-up visits include but are not limited to color Doppler ultrasound, with subsequent advanced imaging using MRI or CT angiography (Table 1).

**Table 1:** Clinical management considerations for brachial plexus compression related to vascular injury.

Considerations for brachial plexus compression related to vascular injury	
Risk factors	Rib fractures, clavicle fractures, shoulder dislocations
Presentation	Decreased or absent pulses, new-onset sensory or motor deficits, pain out of proportion on the examination or increasing in severity
Confounding factors	Acute stress disorder, post-traumatic stress disorder, anxiety, depression, deep vein thrombosis, spinal cord injury
Diagnostic modalities	Duplex ultrasound, computed tomography or magnetic resonance angiography, fluoroscopic angiography
Frequency	Evaluate at frequent intervals following a concerning trauma

## Conclusions

Delayed onset brachial plexus nerve injuries can have a variety of etiologies. Patients with blunt or penetrating cervical or thoracic spinal cord injuries should be closely monitored at follow-up visits for evidence of any sort of vascular or neurological compromise. These patients can develop vascular injuries that can damage local structures including damage to the brachial plexus. Patient education about early warning signs can be coupled with high index of suspicion and early imaging modalities at follow-up visits to ensure timely detection and management.
